# Supplementary material for: Resolving the full spectrum of human genome variation using Linked-Reads
Source: Genome Res. 2019 Apr;29(4):635–45. doi: 10.1101/gr.234443.118 (PMC6442396; doi:10.1101/gr.234443.118)
Supplement: Supplemental Material [file supp_gr.234443.118_Supplemental_longranger-2.2.zip › longranger-2.2.tar.bz2!/lr2.2-public/lib/stan/cmdstan/stan/doc/api/html/sum__values_8hpp_source.html]

Stan: src/stan/common/recorder/sum\_values.hpp Source File


|  |  |
| --- | --- |
|  | Stan  2.5.0  probability, sampling & optimization |


- Main Page
- Related Pages
- Namespaces
- Classes
- Files

- File List
- File Members

All Classes Namespaces Files Functions Variables Typedefs Enumerations Enumerator Friends Macros Pages

- src
- stan
- common
- recorder

sum\_values.hpp

Go to the documentation of this file.

1 #ifndef STAN\_\_COMMON\_\_RECORDER\_\_SUM\_VALUES\_HPP

2 #define STAN\_\_COMMON\_\_RECORDER\_\_SUM\_VALUES\_HPP

3

4 #include <vector>

5 #include <stdexcept>

6 #include <string>

7

8 namespace stan {

9  namespace common {

10  namespace recorder {

11

12  class sum\_values {

13  public:

14  sum\_values(const size\_t N)

15  : N\_(N), m\_(0), skip\_(0), sum\_(N\_, 0.0) { }

16

17  sum\_values(const size\_t N, const size\_t skip)

18  : N\_(N), m\_(0), skip\_(skip), sum\_(N\_, 0.0) { }

19

20

27  void operator()(const std::vector<std::string>& x) {

28  }

29

36  template <class T>

37  void operator()(const std::vector<T>& x) {

38  if (N\_ != x.size())

39  throw std::length\_error("vector provided does not match the parameter length");

40  if (m\_ >= skip\_)

41  for (size\_t n = 0; n < N\_; n++) {

42  sum\_[n] += x[n];

43  }

44  m\_++;

45  }

46

47

53  void operator()(const std::string x) { }

54

59  void operator()() { }

60

64  bool is\_recording() const {

65  return true;

66  }

67

68  const std::vector<double>& sum() const {

69  return sum\_;

70  }

71

72  const size\_t called() const {

73  return m\_;

74  }

75

76  const size\_t recorded() const {

77  if (m\_ >= skip\_)

78  return m\_ - skip\_;

79  else

80  return 0;

81  }

82

83

84  private:

85  size\_t N\_;

86  size\_t m\_;

87  size\_t skip\_;

88  std::vector<double> sum\_;

89  };

90

91  }

92  }

93 }

94

95 #endif

stan::common::recorder::sum\_values::is\_recording

bool is\_recording() const

Indicator function for whether the instance is recording.

**Definition:** sum\_values.hpp:64

stan::common::recorder::sum\_values::operator()

void operator()()

Do nothing.

**Definition:** sum\_values.hpp:59

stan::common::recorder::sum\_values::sum\_values

sum\_values(const size\_t N, const size\_t skip)

**Definition:** sum\_values.hpp:17

stan::common::recorder::sum\_values::operator()

void operator()(const std::vector< T > &x)

Add values to cumulative sum.

**Definition:** sum\_values.hpp:37

stan::common::recorder::sum\_values::called

const size\_t called() const

**Definition:** sum\_values.hpp:72

stan::common::recorder::sum\_values::operator()

void operator()(const std::string x)

Do nothing with a string.

**Definition:** sum\_values.hpp:53

stan::common::recorder::sum\_values::sum\_values

sum\_values(const size\_t N)

**Definition:** sum\_values.hpp:14

stan::common::recorder::sum\_values::sum

const std::vector< double > & sum() const

**Definition:** sum\_values.hpp:68

stan::common::recorder::sum\_values::recorded

const size\_t recorded() const

**Definition:** sum\_values.hpp:76

stan::common::recorder::sum\_values

**Definition:** sum\_values.hpp:12

stan::common::recorder::sum\_values::operator()

void operator()(const std::vector< std::string > &x)

Do nothing with std::string vector.

**Definition:** sum\_values.hpp:27


---

[ Stan Home Page ]

*© 2011–2014,
Stan Development Team.*
